# Population density and group size effects on reproductive behavior in a simultaneous hermaphrodite

**DOI:** 10.1186/1471-2148-11-107

**Published:** 2011-04-18

**Authors:** Dennis Sprenger, Rolanda Lange, Nils Anthes

**Affiliations:** 1 Animal Evolutionary Ecology, Institute of Evolution and Ecology, University of Tübingen, Auf der Morgenstelle 28, 72076 Tübingen, Germany

**Keywords:** population density, mating group size, optimal mating rate, sexual conflict, simultaneous hermaphrodite

## Abstract

**Background:**

Despite growing evidence that population dynamic processes can have substantial effects on mating system evolution, little is known about their effect on mating rates in simultaneous hermaphrodites. According to theory, mating rate is expected to increase with mate availability because mating activity is primarily controlled by the male sexual function. A different scenario appears plausible in the hermaphroditic opisthobranch *Chelidonura sandrana*. Here, field mating rates are close to the female fitness optimum, suggesting that mating activity remains unresponsive to variation in mate availability.

**Results:**

Applying an experimental design that aims at independent experimental manipulation of density and social group size, we find substantial increases in mate encounter rate with both factors, but no statistically detectable effects on mating rate in *C. sandrana*. Instead, mating rate remained close to the earlier determined female fitness optimum.

**Conclusions:**

We demonstrate that mating rate in *C. sandrana *is largely unresponsive to variation in mate availability and is maintained close to the female fitness optimum. These findings challenge the prevailing notion of male driven mating rates in simultaneous hermaphrodites and call for complementary investigations of mating rate effects on fitness through the male sexual function.

## Background

There is increasing awareness that ecological processes such as changes in population demography can substantially affect the evolution of mating systems (reviewed in [1]). For example, theoretical and empirical work shows that female selectivity generally increases at higher population densities, resulting in stronger sexual selection on males [2-8]. Yet, few studies to date have investigated the effect of variation in population density on realized mating rates [1]. In water striders and dung flies, two systems where males are capable of enforcing female matings, empirical work showed that mating rate increases with population density as expected [9,10]. Alternatively, in systems where females have full control over mating probability and mating partners are chosen randomly, theoretical work predicts that mating rates should remain unresponsive to variation in population density [11]. This is because, in the absence of male harassment, females can maintain mating rates close to their reproductive optimum by modulating the number of matings with males accordingly. Yet, empirical investigations of this scenario are lacking to date.

In simultaneous hermaphrodites, the situation is less straightforward than in separate sex species. Mating rates of both sexual functions are often directly linked via reciprocal mating, where individuals donate and receive sperm during a single copulatory bout [12,13]. Theory suggests that, in simultaneous hermaphrodites, mating rates are largely driven by the male sex function for two, reasons. First, similar to many separate sex species and in accordance with Bateman's Principle [14], the male function is thought to show a steeper fitness gain with increasing mating success than the female function ([15,16] but see [17]). Second, individual costs associated with matings in the female function can be compensated by a gain in paternity [18], rendering hermaphrodites likely to accept even costly female matings. If mating rate is indeed controlled by the male function, one would expect that mating rate increases at higher population densities because higher densities should increase mate encounter rates and thus mating opportunities. In support of this prediction a recent study on the hermaphroditic flatworm *Macrostomum lignano *showed that mating activity increased with partner availability [19]. Similarly, the freshwater snail *Lymnaea stagnalis *was found to receive more inseminations when kept in groups of eight compared to groups of four or pairs [20].

A different scenario being concordant with the predictions by Härdling & Kaitala [11] appears plausible for the simultaneously hermaphroditic opisthobranch *Chelidonura sandrana*. In this non-selfing internal fertilizer, multiple matings are frequent in both sexual functions [21]. Experimental work showed that maternal per-egg investment depends on the number of different mating partners [22], being maximized at an intermediate mating rate of 2-3 matings per day [23]. At this mating rate, fecundity costs due to multiple matings appear to be offset through reduced embryo mortality [23,24]. Field surveys further revealed that this intermediate daily mating rate established in the laboratory closely matches realized field mating rates [21]. All these findings suggest that the detected mating rate is close to the female fitness optimum. Moreover, because mate encounter rates typically exceed the number of copulations in this species (personal observations) the natural mating rate may be maintained close to the female fitness optimum independent of mate availability.

Against this background our main objective was to experimentally test for mate availability effects on mating rate in *C. sandrana*. Given that frequent matings in the female function result in reduced fecundity [23] and that comparable studies are rare in simultaneous hermaphrodites, we further tested for mate availability effects on female fecundity. Because mate availability can be both a function of social group size (the total number of interacting individuals) and density (the number of individuals per area) we applied an experimental approach that, in theory, is capable of differentiating between both effects. We hypothesized that (i) variation in mate availability has little effect on mating activity and that (ii) the realized mating rate will be close to the earlier determined fitness optimum whenever mate encounters are sufficiently high to maintain this optimal mating rate. Because our predictions match those of the theoretical work by Härdling and Kaitala [11] developed for separate sex species (see above), we discuss our findings accordingly.

## Results

**Figure 1:**
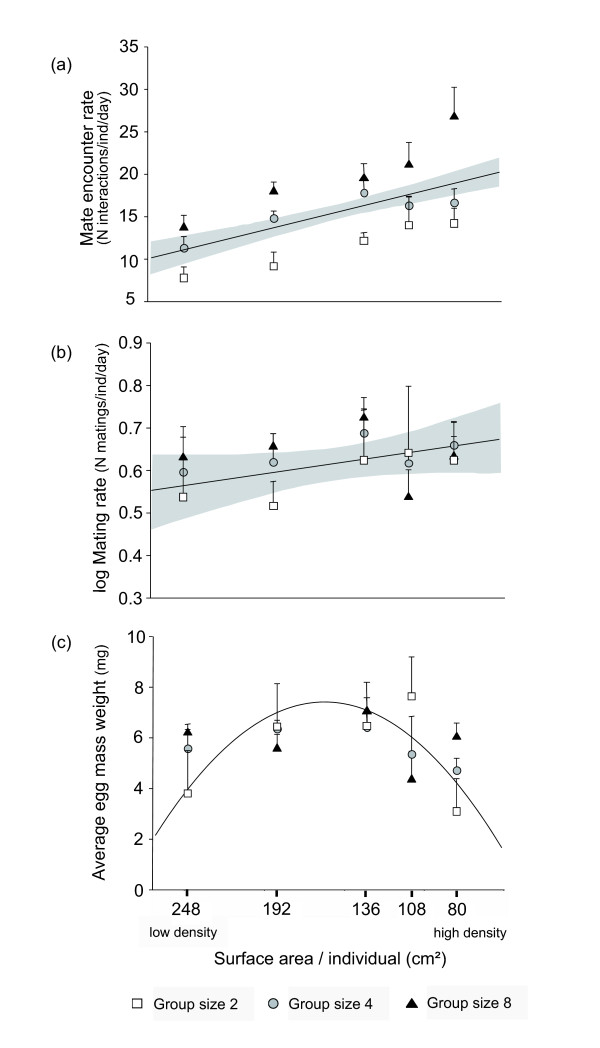
**Effects of density and group sizes on (a) mate encounter rate, (b) individual daily mating rate (log-scale), and (c) average egg mass weight**. Graphs depict raw data (cf. Table 1), with each data point representing the mean ± SE of 5 replicates. Regression lines illustrate the overall effect of density when group sizes are combined, after correcting for variation between experimental runs. Panels (a) and (b) show fitted linear regression lines and their 95% confidence intervals (grey shades, see Material and Methods for details), panel (c) shows the fitted quadratic regression term.

Mate encounter rates strongly increased with both mating group size and population density (Table 1; Figure 1a), with an approximately 2-fold increase each from the lowest to the highest density categories and from the smallest to the largest mating groups. Despite this, the average mating rate per individual and day remained constant across all treatments (mean ± SD: 3.44 ± 1.56; range: 0.57 - 8.86) (Table 1; Figure 1b). Our analysis indicated a statistically weak trend for mating rate to increase with density (P = 0.1, Table 1; upper 95% bound for the corresponding linear regression slope equals 0.5 matings per day, corresponding to a 15% increase at maximum in average mating rate across a 300% increase in density). However, inspection of raw data suggests that this effect was largely caused by reduced mating rates at the lowest two density treatments in group size 2 (Figure 1b), whereas mating rates remained stable across all other treatment combinations. Average egg mass weight per replicate container (mean ± SD in mg: 5.64 ± 0.29) varied significantly with density but not with group size (Figure 1c). The relationship was best explained by a curvilinear regression peaking at intermediate densities (Table 1; Figure 1c). All other parameters were not significantly affected by mate availability (Table 1).

**Table 1:** General Linear Mixed Model results for the effects of group size (nominal factor) and density (continuous factor) on mating behaviour and fitness components.

Parameters	df	Type III SS	*F*	*P*	*R*^2^
Mate encounter rate (per individual & day)					
Full model (linear)	7	3208.68	16.36	**< 0.001**	0.63
Group size	2	1554.93	27.75	**< 0.001**	
Density	1	1134.29	40.49	**< 0.001**	
Run	4	479.22	4.28	**0.004**	
Error	66	1848.92			
					
log Mating rate (per individual & day)					
Full model (linear)	7	4.16	4.05	**< 0.001**	0.30
Group size	2	0.17	0.56	0.57	
Density	1	0.41	2.82	0.10	
Run	4	3.52	6.00	**< 0.001**	
Error	66	9.69			
					
Average egg mass weight					
Full model (quadratic)	8	2347.63	3.60	**0.002**	0.31
Group size	2	4.47	0.03	0.97	
Density	1	112.08	1.38	0.24	
(Density)^2^	1	1189.43	14.61	**< 0.001**	
Run	4	1162.98	3.57	**0.01**	
Error	65	5291.15			
					
Total egg mass weight per individual					
Full model (linear)	7	10019.56	9.21	**< 0.001**	0.49
Group size	2	235.63	0.76	0.47	
Density	1	12.34	0.08	0.78	
Run	4	9772.64	15.72	**< 0.001**	
Error	66	10254.59			
					
*N *egg masses per individual					
Full model (linear)	7	49.08	17.97	**< 0.001**	0.66
Group size	2	0.53	0.68	0.51	
Density	1	0.04	0.11	0.74	
Run	4	48.44	31.03	**< 0.001**	
Error	66	25.75			

Significant P-values are indicated in bold. Note that interaction terms with P ≥ 0.25 were removed from the analysis (see Material and Methods for details).

## Discussion

We found that mate encounter rates and thus mating opportunities increased with both group size and density. Despite this, the average individual mating rate remained largely constant and close to the earlier detected fitness optimum of the female function in *C. sandrana *[23]. Importantly, the observed increase in mate encounters across densities by ~100% did not transform in a statistically detectable increase in mating rate, where the maximum change rendered undetectable due to noise in the data does not exceed a 15% increase across densities (upper 95% CI bound of regression slope). This at best weak effect can largely be attributed to reduced mating activity at the lowest mate encounter rates, indicating that under such conditions optimal mating rates are difficult to maintain, whereas mating rates remain stable at higher densities where mate encounters are sufficiently frequent.

Although the existing variation in mating rate implies strong between-individual differences in mating activity, the documented absence of significant group size and density effects clearly contradicts the general notion of a tight positive relationship between mate availability and average mating rate as previously reported from other simultaneously hermaphroditic [20,19] and separate sex [9,10] species. Instead, our findings indicate that the average daily mating rate in *C. sandrana *is largely independent of mate availability and close to the female fitness optimum, as long as the latter can be realized. These findings challenge the general notion of male driven mating rates in simultaneous hermaphrodites and suggest three alternative scenarios. First, conflict over mating rate may be minute or even absent if both sexual functions share similar mating rate optima, i.e. when the here detected average mating rate is close to the female and male mating optimum. In this case, no sex function would ultimately control matings, but realized mating rates result from mutual interest. Such low male mating rate optima are plausible if remating in the male sexual function generates accelerating costs, e.g. via sperm digestion by the sperm recipient [25]. However, previous work in *C. sandrana *contradicts this scenario of accelerating male costs because animals are easily capable of donating sperm up to eight times within 9 h [22]. Second, realized mating rates may be intermediate between divergent male and female optima while providing roughly similar fitness returns for both sexual functions (representing inherent sexual antagonism;[26]). Under this scenario, mating rate represents the balanced result of opposing sex-specific interests. Our previous finding that realized field mating rates are close to the female fitness optimum [23] do not lend support to this scenario. Third, assuming that the male mating rate optimum exceeds that of the female function (see [27] for conforming data in freshwater planarians), then mating rate should be largely controlled by the female function as it is close to the female fitness optimum of *C. sandrana *[23]. This last scenario is currently most concordant with the available data but detailed information on mating rate effects on male fitness are clearly needed to substantiate this conclusion.

Interestingly, our findings conform to recent theoretical predictions for separate sex species by Härdling & Kaitala [11]. Their model predicts that females evolve constant mating rates that are largely independent of mate availability if the following three central assumptions are met. First, female fitness solely depends on the number of different male partners, not on the individual quality of certain males. Thereby the model explicitly excludes female choosiness for high quality males as an adaptive trait. Second, mating probability is under full female control, excluding systems in which females accept matings due to sexual harassment. Third, female fitness is maximized at an intermediate mating rate at which the fecundity benefits and the mortality costs of multiple mating balance. All these three key model assumptions appear to be met in our study system *C. sandrana*. First, polyandry-mediated benefits primarily depend on the number of different mating partners, not on the identity or quality of mates [22]. The production of multiply sired egg masses seems to represent a diversifying genetic bet-hedging strategy that increases the probability of offspring survival under fluctuating environmental conditions [24]. Female fitness is thus a direct function of mating frequency irrespective of male quality. Second, male acting individuals do not show any harassment or otherwise manipulative behavior of female mating activity [24,28]. This is further supported by the observation that realized mating rates both under laboratory and field conditions [21] are close to the female fitness optimum. Third, female fitness is maximized at an intermediate mating rate, where fecundity costs of multiple matings appear to be offset through increased offspring viability [23].

We further found that fitness measures for the female function were independent of variation in group size and solely a function of density, with fecundity being maximized at an intermediate density. Because mating rate remained constant across all these treatments, density-dependent differences in fecundity are unlikely to represent mere differences in allosperm availability. Instead, reduced fecundity at low densities might represent costs due to increased mate searching. Interestingly, previous work in *C. sandrana *showed that repeated matings with the same male result in decreased fecundity relative to repeated matings with different males [22]. Because the probability of mating with the same partner is higher at lower densities, the same currently unidentified mechanism might contribute to reduced offspring production at low densities. In this context it remains puzzling, however, why group size, i.e. the actual number of available mating partners, had no comparable effect on fecundity in the present study, because the likelihood of copulating repeatedly with the same partner equals 1 in our pair treatment. Perhaps, differences in social group size do not reflect differences in mating group size, with the latter being primarily affected by the actual distance between animals (i.e. density).

Decreasing fecundity at the highest densities may be the result of a shift in sex allocation towards the male function. Although our data provide no direct measure of sex allocation, various studies confirmed rapid strategic reallocation of resources towards a hermaphrodite's male function with increasing social group size (reviewed in [29]). For example, in *Macrostomum lignano*, testis size tends to increase with social group size while ovary size significantly decreases [30]. Although constancy in average daily mating rate may suggest little variation in mean sex allocation in *C. sandrana*, resource allocation may have been adjusted in response to mate availability rather than to mating rate. Unlike in *M. lignano*, however, sex allocation cannot be measured *in vivo *in *C. sandrana*, rendering definite conclusions on sex allocation adjustment difficult. Possible components of resource re-allocation that should be explicitly addressed in follow-up studies include the amount of sperm or seminal fluid transferred, the composition of these seminal fluids (e.g. with respect to manipulative substances, [31]), or energetic investment in other male components such as precopulatory interactions, all of which may contribute to compromised female fecundity at high densities. Finally, declining fecundity at high densities may have been caused by waste products or certain metabolites, which have been suggested to act as egg laying inhibitory substances in pulmonate gastropods [32]. Although water was regularly exchanged and experimental containers were frequently cleaned to reduce such effects, it is possible that inhibitory substances accumulated at higher densities or were actively produced by animals in response to higher mate encounter rates.

The here documented differential effects of group size and density on reproductive behaviour imply that studies on mate availability effects need to carefully disentangle both factors by applying an appropriate experimental design. However, our findings also indicate that a non-random distribution of animals over the available space may make a clear differentiation between the relative contributions of group size and density on mating opportunities difficult, even though the applied experimental design allows, in theory, to do so. For example, while mate encounter rate would be expected to increase at higher densities as the distance between individuals is reduced [1], mate encounter rates in our study also increased with group size independent of density. Given that mating aggregations are prevalent in many animal systems, future experimental work that directly manipulates spatial distribution is needed in order to shed light on the detailed relationship between aggregation behavior and reproductive behavior.

## Conclusions

Contrary to classic predictions by theory, we here show for the first time that mating rate is largely unresponsive to variation in mate availability in a simultaneous hermaphrodite. With mating rates being close to the female fitness optimum, our findings challenge the prevailing notion of male driven mating rates in simultaneous hermaphrodites and call for complementary investigations of mating rate effects on fitness through the male sexual function.

## Methods

### Study species

*Chelidonura sandrana *Rudman 1973 (Cephalaspidea, Aglajidae) inhabits tropical marine shallow water sand flats. Copulations are unilateral, but sexual roles are usually alternated during a copulatory bout, resulting in reciprocal sperm exchange (details in [28]). Following mating, egg masses containing hundreds of singly encapsulated eggs are deposited on the substrate every few days [22]. Embryos develop into feeding veliger larvae with a planktonic period of unknown length. Animals are sexually active throughout the Australian summer (personal observation) and occur in patchy aggregations with up to dozens of individuals/m^2^.

### Sampling and maintenance

Individuals were collected in 0.5-8 m depth around Lizard Island, Queensland, Australia, in December 2007 and January 2008 (permit G05/15308·1 of the Great Barrier Reef Marine Park Authority). On the day of collection, body wet weight was measured to the nearest mg. Animals were then randomly distributed among the experimental groups and kept at 26°C water temperature and a natural diurnal cycle. Water was stirred twice per day and experimental containers were thoroughly cleaned and refilled with fresh seawater every fourth day to minimize accumulation of waste products. Every other day animals were fed with their natural food source, the flatworm *Wulguru cuspidata *Winsor, 1988 ad libitum.

#### Experimental setup

**Figure 2:**
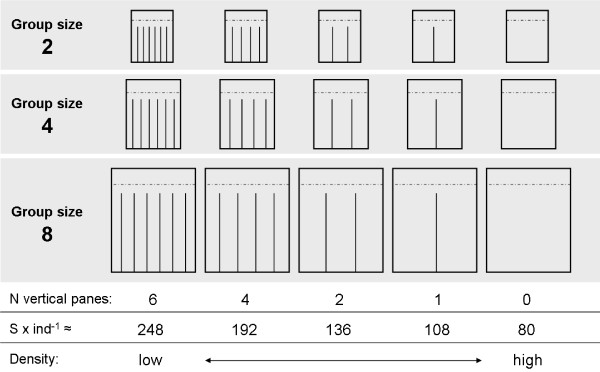
**Schematic presentation of experimental tanks (lateral view) for the three group sizes**. Note equal surface areas (S) per individual (in cm^2^) within group sizes. Dashed horizontal lines indicate the water level. Vertical panes are indicated by vertical lines. Note that water volume per individual was kept constant at 612 ml by placing each experimental tank into a larger standardized water tank (not shown).

In order to independently manipulate density (5 treatments: 80 to 248 cm^2 ^surface area per individual; Figure 2) and group size (3 treatments: 2, 4, and 8 individuals per container; Figure 2) we built 15 customized experimental containers using 3 mm acrylic glass and Acrifix^® ^120 (Seven Hills, Australia) acrylic glue. Because movements of *C. sandrana *are restricted to the benthic substrate, i.e. a 2-dimensional space with equal usage of vertical and horizontal planes (personal observation), we only varied surface area per individual (see below), but not the water volume (612 ml per individual across all treatments) by placing each experimental container into a larger standardized water tank. Water exchange was assured by replacing one of the side walls of the experimental tanks with fine mesh. Manipulation of surface area within group sizes was achieved by placing 0, 1, 2, 4, or 6 acrylic vertical panes into each of 5 experimental containers, respectively (Figure 2). Data were collected over 5 experimental runs, with each run lasting 11 days (4 days of acclimation and 7 days of data collection). When transferring individuals to their assigned containers they were distributed evenly in space. Within each run we performed 5 replicates per group size, i.e. one for each density treatment per group size, resulting in a total of 25 replicates for each group size and 15 replicates for each density. One replicate (group size 2 with 1 divider) had to be excluded from the analysis due to the death of one animal.

#### Behavioral and fitness measures

Each container was observed 3 times per day (8:00, 12:00, 16:00) for 30 minutes and the following parameters were recorded: (i) *Mate encounter rate*: Because behavioral activity ceases at night (personal observation) we assumed a daily activity period of 12 h, i.e. 8 times the daily observation period of 1.5 h. Daily mate encounter rate per individual thus calculates as 8*((N observed mate encounters per container * 2 individuals/7 observation days)/group size). (ii) *Mating rate*: an estimate of the average number of copulations for each individual per day. Given that this species typically alternates sexual roles within a copulatory bout [33] the obtained mating rates can be easily transformed into estimated numbers of matings in both sex roles. Considering a daily activity period of 12 h (see above), individual daily mating rate calculates as 8*((N observed matings per container * 2 individuals/7 observation days)/group size). Matings that were already in progress at the start of observations or that continued beyond the observation period were weighted by 0.5 to avoid artificial inflation of mating rate estimates. Assuming that an individual would rarely be involved in more than one copulation per 30' observation interval, with average copulation duration ~10 min ([28,34]), our observation scheme allowed detecting individual daily mating rates ranging between zero and 24.

For fitness measurements, egg masses were collected daily from each container and the following parameters were recorded: (i) *Mean egg mass weight per container*: Total egg mass weight per container divided by the number of egg masses; (ii) *Total egg mass weight per capita*: Total egg mass weight per container divided by the number of individuals; (iii) *N egg masses per individual*: egg mass count per container divided by the number of individuals.

### Statistical analysis

We used a General Linear Mixed Model to test for the effects of group size (nominal fixed factor) and density (continuous fixed factor) on female reproductive parameters as dependent variables. All dependent variables were entered as means per experimental container, and their values were log-transformed if necessary to meet the assumptions of normality in data and residual distributions. To account for temporal effects, we included the term experimental run as a random effect. In order to assess whether density effects varied between the three group sizes, we further included the interaction group size * density as a fixed factor. The interaction term was omitted from the final models whenever *P *exceeded 0.25 [35]. Larger groups typically yield less variable estimates of traits than smaller groups, therefore violating the assumption of equality in variances. To meet requirements of parametric testing, we thus weighted each dependent variable differentially for each group size [36]. Weighting factors were 1 for group size 8, 2 for group size 4, and 4 or multiples of 4 for group size 2, until no significant differences in variance (Levene's test) were detectable between groups [36]. In cases where visual inspection indicated a non-linear effect of density on mean egg mass weight, we added density as a second-order polynomial term and tested whether the reduced or the full model provided a better fit using partial *F *statistics [36]. For near significant parameters (0.05 ≤ *P *≤ 0.1) we calculated the upper bound of the 95% confidence limits for the regression slope over the complete density range (representing a 300% increase from the lowest to the highest density) to quantify the maximum undetected change across the corresponding predictor variable range. All statistical analysis were performed with JMP IN version 8·0·1 (SAS Institute Inc., Cary, NC, USA).

## Authors' contributions

DS and NA designed the experiments. DS and RL collected and analyzed the data. DS wrote the manuscript and NA and RL corrected earlier drafts. All authors read and approved the final draft of the manuscript.
